# Blockchain-enabled identity management for IoT: a multi-layered defense against adversarial AI

**DOI:** 10.1038/s41598-026-35208-y

**Published:** 2026-02-02

**Authors:** Muhammad Usama, Arshad Aziz, Nada Alasbali, Nazik Alturki, Muhammad Hanif, Mujeeb Ur Rehman

**Affiliations:** 1 Department of Cyber Security, Pakistan Navy Engineering College, National University of Sciences and Technology (NUST), Karachi, 75350 Pakistan; 2 Department of Computer Science, Main Campus, Iqra University, Karachi, 75500 Pakistan; 3 Department of Informatics and Computer Systems, College of Computer Science, King Khalid University, Abha, 61421 Saudi Arabia; 4 Department of Information Systems, College of Computer and Information Sciences, Princess Nourah bint Abdulrahman University, P.O. Box 84428, Riyadh, 11671 Saudi Arabia; 5 Department of Informatics, School of Business, Örebro Universitet, Örebro, Sweden; 6 School of Computer Science and Informatics, De Montfort University, Leicester, LE1 9BH United Kingdom

**Keywords:** Internet of Things (IoT), Blockchain, Smart Contracts, Zero-Knowledge Proofs, Adversarial Machine Learning, Identity Management, Engineering, Mathematics and computing

## Abstract

The growing deployment of the Internet of Things (IoT), especially in critical infrastructure, has increased the need for identity systems that are scalable and robust against attacks. However, existing centralized systems have fundamental weaknesses, especially where adversaries use artificial intelligence (AI)-based techniques, such as generative spoofing, model poisoning, and deepfakes to create fake identities. In this paper, we present a novel blockchain-based IoT security system that combines decentralized identity verification, zero-knowledge proofs, Byzantine-resistant federated learning, and formal verification of smart contracts. The proposed architecture eliminates single points of trust, allows device registration while preserving privacy, and provides defense against AI-driven attacks through formally modeled state transitions. Experimental results show that this method shows significant improvements over previous frameworks, including a 48% reduction in false acceptance rate during GAN-based spoofing and speedup the ZKP verification. This work provides a blockchain-enabled identity management system for IoT to encounter AI-based threats and maintain a balance between performance and security with the help of adversarial simulation, symbolic execution, and threshold cryptography.

## Introduction

According to recent security reports, about 83% of IoT systems are still vulnerable to advanced attacks, including adversarial spoofing, model poisoning, and deepfake-based identity spoofing^1,2^. Centralized structures such as certificate authorities and platforms often prove to be single points of failure, making it easy to commit large-scale identity theft, denial-of-service (DoS) attacks, and unauthorized identity takeovers. In addition, the increasing use of generative adversarial networks (GANs) has led to an increase in biometric spoofing attacks, which defeat traditional authentication methods at a much higher rate^3,4^.

Moreover, with the emergence of modern IoT security architectures, several new and very sensitive challenges have emerged. First, centralized institutions such as traditional public key infrastructure (PKI) and certificate authorities act as a single point of failure. If one of these institutions is compromised, the effects can spread throughout the system in the form of large-scale identity theft^2,5^. Second, the scope of threats has expanded significantly since artificial intelligence-based attacks, especially advanced generative models, have shown the ability to generate almost flawless fake signals in attacks such as biometric spoofing^3,6^. Third, empirical evidence suggests that a large number of Ethereum-based smart contracts have vulnerabilities that can be easily exploited by adversarial perturbations created by AI agents^7^.

To address these issues, this paper presents a *Blockchain-Enabled IoT Security System* that uses formal mechanisms, cryptographic techniques, and decentralized smart contract logic to make the device identification system secure, verifiable, and robust against adversarial attacks.

## Related work

The convergence of blockchain, IoT security, and AI-protected authentication has generated a wide range of work across various research communities. This section provides a critical review of current research, divided into four main categories: decentralized identity (DID) frameworks for IoT, adversarial resistance in federated learning, formal authentication of smart contracts, and behavioral biometrics for user interface (UI) security.

### Decentralized identity in IoT

Traditional centralized identity systems are now widely viewed as inadequate for IoT platforms, as they are highly dependent on trusted third authorities with limited scalability. Any failure or security breach at the central authority can disrupt the entire IoT ecosystem. To address these issues, blockchain-based decentralized identity (DID) models^8^ have emerged, which provide self-sovereign identity (SSI) and verifiable credentials. Initially, Sovrin and uPort introduced the concept of DID by enabling identity ownership and transfer. Later, Dorri et al.^9^ presented a lightweight blockchain architecture for IoT systems to reduce computational burden. However, it does not provide strong formal security mechanisms against identity spoofing or Sybil attacks.

Fan et al.^10^ improved access control and privacy by incorporating verifiable credentials through DIAM-IoT. Similarly, Waleed and Kosta^11^ presented a comprehensive review of DID systems in IoT, but their research does not consider adversarial attacks arising from generative AI. Blockchain-based decentralized PKI systems (DPKI) are also becoming increasingly popular. Papageorgiou et al.^12^ proposed a blockchain-based PKI system that eliminates the single point of failure problem. However, most DPKI solutions do not natively provide zero-knowledge proof (ZKP). Panigrahi et al.^13^ improved the auditability and tamper resistance of PKI by incorporating smart contracts within it, but their work has not yet been fully tested against attacks such as deepfake-based credential fraud.

### Adversarial robustness in federated learning (FL)

Federated learning (FL) enables collaborative model training across distributed nodes without sharing raw data^15^. This decentralized approach, while having practical advantages, is also quite susceptible to adversarial attacks, especially model poisoning^16^. To address this issue, Blanchard et al.^14^ introduced the Krum algorithm, which is a robust aggregation method and isolates malicious or irregular gradients. Other approaches include statistical filters such as Trimmed Man and Bulyan that attempt to reduce adversarial effects.

In recent work, Yazdinejad et al.^17^ proposed a privacy-preserving FL framework based on homomorphic encryption, which provides better resistance to poisoning attacks. Similarly, Xia et al.^18^ presented a detailed review of the types of poisoning attacks in FL and the existing defense techniques against them. Colosimo and De Rango presented the method of dynamic gradient filtering^19,20^, while Daukantas et al.^21^ worked on improving statistical robustness through formal verification. Sasaki and Ura^22^ developed estimators that can maintain performance despite trimming bias in contaminated datasets. Furthermore, Baniecki and Biecek^23^ examined adversarial vulnerabilities in explainable AI (XAI), and Wortsman et al.^24^ presented proxy-based diagnostic methods for identifying instability of large transformer models.

Although these techniques are effective in experimental environments, they are often inadequate against attacks that use adaptive or changing distortions generated with the help of artificial intelligence. Therefore, Krum was chosen for its ability to effectively exclude irregular gradients and provide resistance to poisoning attacks, while performing reliably in dynamic environments with minimal tuning.

### Formal verification of smart contracts

Smart contracts are very useful to automated execution in blockchain systems, but they may lead to logic errors and exploits. Durieux et al.^27^ conducted a comprehensive review by considering Ethereum smart contract analysis tools and showed that many smart contracts used in practice contain serious security vulnerabilities. Smaragdakis et al.^28^ performed the symbolic value analysis by modeling Ethereum-based smart contract logic with greater depth and precision and presented the serious security flows. In another work, Singh et al.^25^ reviewed formal methods and presented two major issues in smart contracts: (1) The lack of interoperability and standardization in smart contract verification tools that cause serious issues to test smart contracts on different platforms. (2) Scalability and performance issue of the complex or large smart contracts with existing formal methods. Similarly, Tolmach et al.^26^ identified some notable issues by performing detailed classification of the formal specification languages and verification tools: (1) The expressiveness of existing languages is limited that cause the serious issue to fully understand the complex logic of the smart contracts. (2) The verification chain is often fragmented which required different tools on different stages; therefore, it makes the entire process complex, time-consuming, and error-prone. Proposed study employed the Manticore, a symbolic execution engine, to systematically analyze the logical correctness, state transitions, and all possible execution paths of the smart contracts to effectively identify potential vulnerabilities and ensure that smart contracts behave as expected.

### Behavioral biometrics and deepfake resistance

Behavioral biometric methods are gaining popularity to verify a user’s identity as compared to the conventional passwords and physical biometrics methods. Behavioral biometric analyzes the user behavior by considering user’s typing style, screen touch patterns, and specific movement patterns and distinguish a genuine user from a potential attacker. In^29^, Ellavarason et al. reviewed the behavioral biometric systems that runs on mobile platforms and found the tradeoff between usability and performance by identify that simpler systems have higher the accuracy rate. In^30^, Mhatre et al. studied how behavioral profiles change in the context of cybersecurity, and observed that AI-powered deepfake technology creates new and complex threats. Furthermore, Qin et al.^31^ proposed an explainable artificial intelligence (XAI) technique for detecting adversarial tampering in behavior-based authentication, while Zhan et al.^32^ presented an anomaly detection model that identifies fraud patterns by learning the user’s key behavioral units.

### Synthesis and gaps

Table 1 presents a comparative review of the major research works on DID, FL robustness, smart contract verification, and behavioral biometrics. The comparison was mainly based on how scalable each method is, how effective it is in resisting various attacks, how interoperability it is with existing IoT infrastructure, and what level of security guarantees it provides. Specifically, this study evaluated each method based on execution time, throughput, and resistance to various attack models. Despite significant progress in improving the robustness of DID, FL, smart contract-based authentication, and behavioral authentication, most of these methods operate in isolation. To fill this gap, proposed work presents a blockchain enabled identity management system with multi-layered defense against adversarial AI that integrates formal authentication, secret identity management, and attack-resistant learning to provide secure and robust foundation for IoT systems. The integration of DID, FL, and smart contracts includes several key features such as, DID enables secure device identification without revealing sensitive information; Smart contracts allow users and biometric authenticated devices to interact and automatically enforce the strong access control.

**Table 1:** Comparative analysis of the related work.

Domain	Representative Works	Key Contributions	Limitations	Relevance to Proposed Framework
**DID in IoT**	Fan et al. (2020)^10^, Papageorgiou et al. (2020)^12^, Panigrahi et al. (2023)^13^	Use of DIDs, smart contract-based PKI, privacy enhancement via blockchain	Lack of integration with AI-resistant mechanisms; limited real-time scalability	Supports privacy-preserving identity; basis for blockchain-based authentication
**Adversarial Robustness in FL**	Yazdinejad et al. (2024)^17^, Colosimo et al. (2024)^19^, Daukantas et al. (2021)^21^	Homomorphic encryption, dynamic gradient filtering, robust estimation techniques	Insufficient defense against adaptive attacks and deep poisoning strategies	Core for secure AI model aggregation and verification in IoT
**Formal Verification of Smart Contracts**	Smaragdakis et al. (2021)^28^, Singh et al. (2020)^25^, Durieux et al. (2020)^27^	Symbolic/static analysis, vulnerability scans on large-scale contracts	Often underutilized in IoT; not resilient to AI-manipulated contract logic	Formal foundation for secure device interactions and access control
**Behavioral Biometrics and Deepfake Resistance**	Ellavarason et al. (2020)^29^, Qin et al. (2024)^31^, Zhan et al. (2023)^32^	Continuous authentication, XAI for adversarial defense, behavior sequence learning	Deepfake resistance not fully addressed in large-scale settings	Augments non-intrusive user validation under adversarial threats

## Blockchain-enabled IoT security system using ethereum framework

The proposed system presents a blockchain enabled identity management system for IoT with multi-layered defense against adversarial AI. The proposed system consists of five components, as detailed in following subsections to ensure integrity and trust within the IoT ecosystem, while mitigating common AI threats. We assume that IoT devices have sufficient computational resources to perform cryptographic operations such as ZKP and digital signatures. In addition, we assume a reliable network with moderate latency and bandwidth to support communication between IoT devices and validators.

### Decentralized IoT device registration and authentication

The proposed system starts with the registration of IoT devices, where each device must pass through identity verification process and prove authenticity before participating in the blockchain transactions. The identity verification process guarantees that only authenticated IoT devices are registered in blockchain network. Moreover, aligning the FSM with the identity verification process provides a fundamental and secure progression from device ’Registation’ to ’Transaction’ validation, as illustrated in the table. 2.

**Table 2:** FSM states and testing phases.

FSM State	Testing Phase
Registration	Device Onboarding Test
Verification	Biometric Authentication Test
Transaction	Secure Transaction Test
$F = $ Testing Success	Local Testing and Validation

In registration process, each IoT device $D_i$ generates key pair of public and private keys $(pk_i, sk_i)$. The public key $pk_i$ is then hashed using one-way cryptographic hash $H(pk_i)$ to create device identity and submitted to the deployed smart contract on the blockchain for registration. Once the device identity is registered, it becomes immutable that cannot be changed or deleted. Therefore, this process provides protection from unauthorized access and enables verifiable device credentials. The process is formally defined as follows:

1 $$\begin{aligned} \mathcal {S} = (Q, \Sigma , \delta , q_0, F) \end{aligned}$$

where, *Q* represents the set of system states $\{Registration, Verification, Transaction, Testing\}$, $\Sigma$ represents the set of input symbols $\{DeviceID, SmartContractCall, UI_{Interaction}, Tx_{Signature}\}$, the transition function $\delta$ defines how the system move between two states, for example $\delta (Registration, DeviceID) \rightarrow Verification$, the initial state $q_0$ is given by $q_0=Registration$, while the *F* represents final state, for instance $F=Testing Success$ indicates successful completion of the end-to-end identity verification process. The transition function $\delta$ is implemented in the simulation environment using state transition logic defined by the input symbols $\Sigma$. During the simulation, we control $\delta$ by tracking all states of the system *Q* and their transitions to ensure the expected behavior of the system under different input conditions. An automaton-based abstraction ensures that all formal verification steps of the IoT device registration process are performed in the correct order. The system implements principle of immutable identity binding for each registered device to ensure the integrity and uniqueness:

2 $$\begin{aligned} H(pk_i) \rightarrow \text {stored on-chain} \end{aligned}$$

with the security property:

3 $$\begin{aligned} \not \exists \ pk_j \ne pk_i \ \text {such that} \ H(pk_j) = H(pk_i) \end{aligned}$$

This ensures that two different devices cannot generate the same hash code, thereby eliminating the possibility of collisions or identity forgery. The proposed system integrates the liveness detection and consensus-based validation to prevent attackers from penetrating the system using GAN generated or simulated devices:

1. **Liveness Detection:** To determine liveness, each device sends a biometric signal $B_i$, such as an electromyogram or electrocardiogram, for comparison with registered biometric templates. The system makes a decision to reject the registration attempt based on a specified threshold if the correlation is below the specified threshold:
  4 $$\begin{aligned} \text {Corr}(B_i, B_{\text {historical}}) < \tau _{\text {live}} \end{aligned}$$
  The liveness detection process ensures that only physically present devices can complete the registration process.
2. **Consensus-Based Validation:** To introduce a distributed layer of trust, a committee of validator nodes collectively evaluates each request of the device registration. This process is implemented using a ZKP, where the device must prove that it possesses the private key $sk_i$ corresponding to the hash $H(pk_i)$ of the public key $pk_i$:
  5 $$\begin{aligned} \Pi _{\text {ZK}} :\text {Prove} \left( \exists \ sk_i \ \text {s.t.} \ H(pk_i) = \text {storedHash} \right) \end{aligned}$$

After the consensus verification process, each validator node submits a binary vote to the system:

6 $$\begin{aligned} \text {Vote}_{\text {reg}} = {\left\{ \begin{array}{ll} 1 & \text {if } \text {ZKP-Verify}(\Pi _{\text {ZK}}) \\ 0 & \text {otherwise} \end{array}\right. } \end{aligned}$$

The device registration is accepted only if a majority of validators successfully verify the ZKP by ensuring the consensus in presence of *f* malicious participants to meet the equation 7 criteria.

### Smart contract framework for trustless identity verification and lifecycle management

Smart contracts play a important role as decentralized and trusted agent in managing all key events on the blockchain, including device registration, identity verification, revocation, and status updates to ensure the security and integrity of IoT devices. Each smart contract ensures device identity integrity and authenticity through the use of cryptographic hash functions and ZKPs, thereby allowing only registered and verified devices to access the system.

These smart contracts are modeled as deterministic components in an FSM; their output is completely predictable on given a set of inputs. They react to events like *SmartContractCall* and $Tx_{Signature}$, which, if satisfied produce transitions between the states. After registering a device, the system submits the hash of the device’s public key $H(pk_i)$ to the blockchain to satisfy the condition specified in equation 3.

This means that every single IoT device is provided with an exclusive, unalterable identity that cannot be faked or cloned. Besides, to maximize the dependability and fault tolerance of the proposed system, the smart contracts run in a Byzantine Fault Tolerant (BFT) condition. In such an arrangement, the security of the consensus is preserved as long as the number of the validators *N* satisfies the condition, as given below:

7 $$\begin{aligned} N \ge 3f + 1 \end{aligned}$$

where, *f* refers to the number of malicious validators, whose presence can still be tolerated by the system to guarantee reliably executed smart contracts even one-third of the participating nodes are compromised. Moreover, the smart contracts incorporate sophisticated validation logic to safeguard from AI-based threats. For example, gradient auditing is used to detect suspicious updates in a smart contract by checking the $L_2$ normality condition for any model weight gradient $\nabla W$:

8 $$\begin{aligned} \Vert \nabla W\Vert _2 \le \epsilon \end{aligned}$$

where $\epsilon$ is a given upper bound limiting the volume of malicious updates. Ultimately, the proposed system implements a proof-of-learning (PoL), denoted as $\text {SC}_{\text {FL}}$, which is intended to verify that the device is indeed participating in the learning process. Formally, it can be defined as follows:

9 $$\begin{aligned} \text {SC}_{\text {FL}} :\text {Verify}(\pi , \nabla W) \end{aligned}$$

where, $\pi$ indicates the ZKP that a gradient has been accurately computed, implying that attackers cannot add harmful updates or AI-generated data to the FL pipeline. As a result, the smart contracts which have been proposed in this research work are both mathematically validated and proved secured in offering robust protection against AI-driven threats.

### Decentralized user interface (UI)

The user interface is a critical factor in delivering secure and user-friendly interactions between the users and the blockchain infrastructure. It allows for the most basic operations like connecting IoT devices, real-time status checking, and searching for cryptographic credentials using access management. Here, UI interactions are recorded as part of the input set $\Sigma =\{DeviceID,SmartContractCall,UI_{Interaction},Tx_{Signature}\}$ in the FSM model. Each state transition, such as going from ’Verification’ to ’Transaction’, is initiated based on inputs generated by the UI and results in signed transactions being sent to smart contracts. Each transaction generated by the UI is cryptographically signed with the user’s private key to maintain the integrity and authenticity.

Behavioral biometrics is incorporated into the system as a proactive security measure to defend against AI-driven threats, such as deepfake UI phishing. By analyzing small interaction patterns such as mouse dynamics and keystroke behavior, the system generates a behavioral profile $U_{\text {genuine}}$ for each legitimate user. During any given session, the similarity score between the live behavior *U* and the saved profile is calculated using the cosine similarity:

10 $$\begin{aligned} \text {Score} = 1 - \text {CosineSim}(U, U_{\text {genuine}}) \end{aligned}$$

The system enables Multi-Factor Authentication (MFA) or completely block the access, in case if this score falls below a predefined threshold by indicating potential impersonation or cloning of the user interface. The probabilistic authentication method is incorporated into the state transitions of the UI, thereby making all the interactions with the front-end more trustworthy.

In addition, the UI is constantly aware of the state because it is monitoring all the events on the blockchain. For example, when the device goes from the *Registration* state to the *Verification*, the UI is monitoring the events the blockchain is sending and is updating the user’s view to show the right system status. In this way, the UI acts like a state-aware component in the FSM, guaranteeing user-friendliness and accessibility at the same time. Moreover, it combines the use of cryptographic provocations and anomaly detection with the user-interaction level to counteract deepfake-phishing attacks and keep the trust in a decentralized environment.

### Secure wallet authentication and transaction control

The Digital Wallet (DW) identifies and controls transactions in the proposed blockchain-based identity system with the help of a cryptographic primitives. The browser-based DW is responsible for two major functions; it enables users to manage their private keys locally and securely sign transactions on the blockchain without disclosing any sensitive information. A transaction that is initiated either through the UI or by the back-end system first goes to the DW where it is signed with the user’s private key and then sent to the blockchain network. Hence, the DW operates as a secure gateway on the basis of non-repudiation and message integrity.

In the proposed system FSM $\mathcal {S} = (Q,\Sigma ,\delta ,q_0,F)$, the DW connects with the inputs $\Sigma =\{DeviceID, SmartContractCall, UI_{Interaction}, Tx_{Signature}\}$, mainly through the $Tx_{Signature}$, which confirms the transitions that are linked to the critical operations. The DW plays the role of a validation layer, making sure that the transition of states like $Verification\rightarrow Transaction$ or $Transaction\rightarrow Testing$ is cryptographically valid in every respect. Additionally, the system employs two main security tactics to avoid AI-driven threats, especially in the case of fraudulent transactions: (1) threshold signatures are utilized to secure vital transactions, such as device revocation or credential updates, by only letting the transaction go through if a predetermined number of trusted signers *t* provide the valid partial signatures, as given below:

11 $$\begin{aligned} \text {Sig}_{\text {req}} = \sum _{i=1}^t \text {Sig}(sk_i) \end{aligned}$$

The proposed system guarantees that, even in cases where access to a wallet by a attacker has been compromised, no high-value transactions can take place, thus preventing AI-driven threats from easily executing their plans unilaterally; (2) One of the features of the DW is the incorporation of artificial intelligence that recognizes anomalies as a module strictly monitoring the behavior of gas fees. By using long-short-term memory (LSTM) models that have undergone training with past transaction data, the system arrives at the mean $\mu _G$ and standard deviation $\sigma _G$ of the gas fee *G*. For a transaction to be considered suspect, it has to meet the following criteria:

12 $$\begin{aligned} \text {Flag if } |G - \mu _G| > 3\sigma _G \end{aligned}$$

The dynamic nature of the detection brings about the system’s ability to uncover and classify any suspicious activities in real time, ranging from adversarial threats generated by AI bots, gas fee manipulation to transaction flooding, all without the need for human intervention. By utilizing these methods, DW transforms into a not only monitor of the underlying system but also a major strategic force in the defense of the system. The inclusion of DW in a FSM guarantees transitions between states in which the data is stored securely, protected by cryptography, and impervious to AI-related cyber threats. In that way, DW is a necessary factor in the trustworthiness, traceability, and uninterrupted operation of the proposed blockchain-based identity verification methods.

### Simulation and verification environment

The simulation and verification environment are pivotal components that guarantee secure and auditable development of the blockchain-based system. This environment renders a native blockchain that mirrors real-world network conditions but does not pose any risks related to the mainnet deployment. Developers can deploy, test, and debug smart contracts in a deterministic and transparent environment as the simulation offers full control over essential blockchain parameters like gas costs, block mining intervals, and event triggers.

In the formal model of the system $\mathcal {S} = (Q, \Sigma , \delta , q_0, F)$, the testing environment is defined as the final state $F = \text {Testing Success}$ , ensuring that all previous state transitions, namely Registration, Verification, and Transaction, are fully validated before moving to the mainnet. Inputs such as $\Sigma = \{SmartContractCall, Tx_{Signature}\}$ are simulated and evaluated to ensure that they operate within defined behavioral boundaries and conform to system invariants. Only after reaching this final validated state can a contract be considered safe for live deployment.

Two robust security mechanisms are used during the testing phase to protect against AI-based threats that targets smart contract vulnerabilities. First, the Manticore tool is used for formal verification to mathematically prove that the smart contract preserves security changes across all accessible states as follows:

13 $$\begin{aligned} \forall s \in \text {SC}_{\text {states}}, \phi (s) = \text {True} \end{aligned}$$

Where $\phi (s)$ denotes a set of logical statements that encode the desired security and correctness properties. This guarantees that the execution path of any adversarially altered logic will still maintain the contract’s defined semantics.

Conversely, the testing framework integrates fuzz testing, which mimics AI generated adversarial inputs through gradient-based perturbations. The focal areas of our targeting are critical smart contract functions such as device registration, identity verification, and transaction processing. As a result of our method, we have unearthed weaknesses of the nature of reentrancy and denial-of-service attacks. The perturbation strength $\eta$ is modified based on the particular attack scenario and the defender’s risk tolerance. If for instance it is a high-risk scenario, $\eta$ may be increased to imitate the assailants who are more aggressive. Using techniques derived from adversarial machine learning, the input is produced as follows:

14 $$\begin{aligned} X_{\text {adv}} = X + \eta \cdot \text {sign}(\nabla _X J(X, y)) \end{aligned}$$

where *X* is a valid input, *J* is a loss function that reflects the deviation from the expected behavior, and $\eta$ is the one that determines how much of the perturbation will be applied. This strategy brings the smart contracts into contact with a wide range of and almost limit cases of inputs and thus, the vulnerabilities that remain hidden in the context of normal testing are uncovered.

Integrating a local simulation and verification environment into a state machine-based architecture guarantees that every smart contract is thoroughly adversarial-tested prior to its actual usage. The system handles the process to reach the final state *F* very quickly and also certifies the system’s performance and flexibility by simulating both regular and malicious interactions. This process is critical for guarding the blockchain infrastructure against AI-driven threats and for the overall robustness of the smart contracts that have been deployed in real-world adversarial environments.

**Listing 1:**
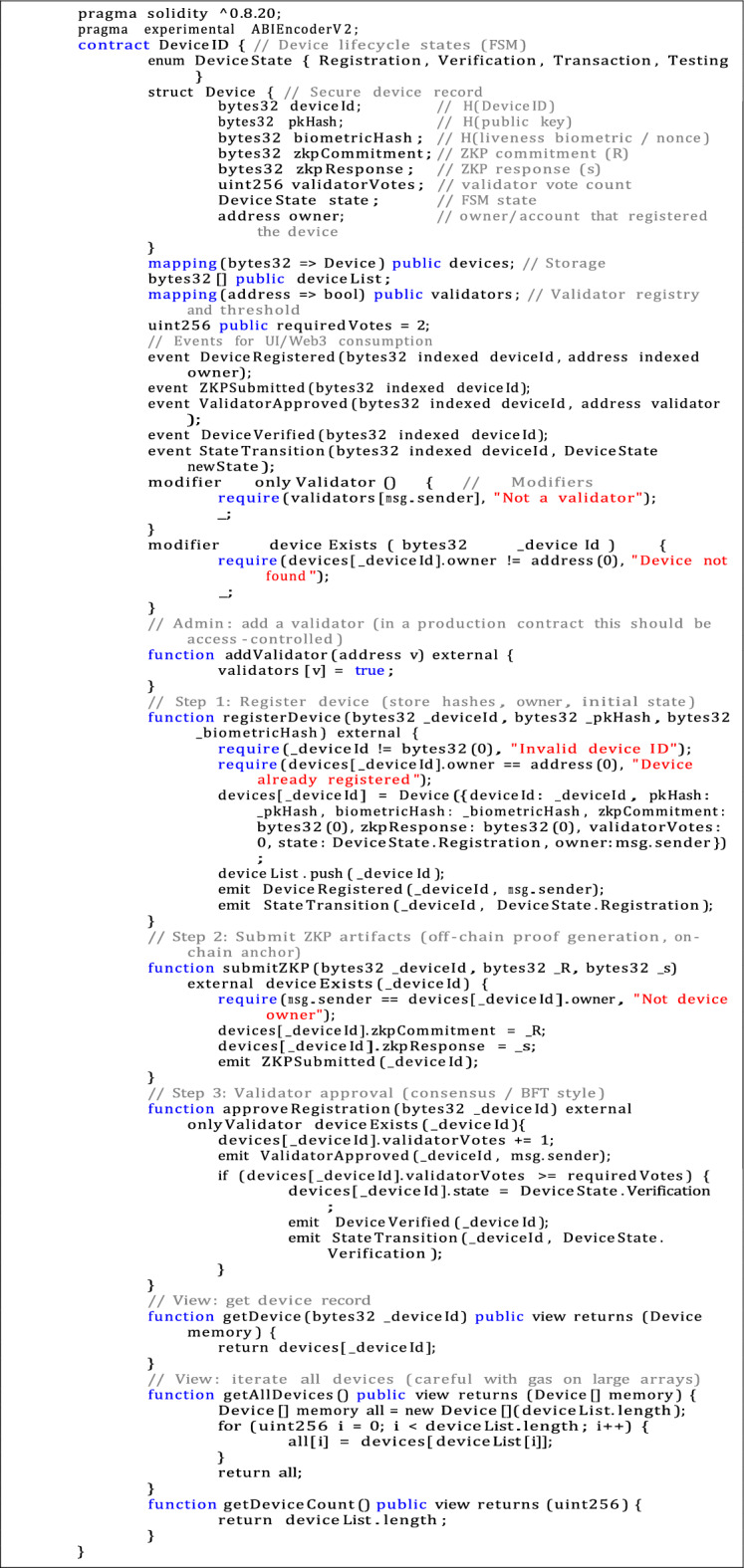
Solidity source code for DeviceID smart contract.

**Listing 2:**
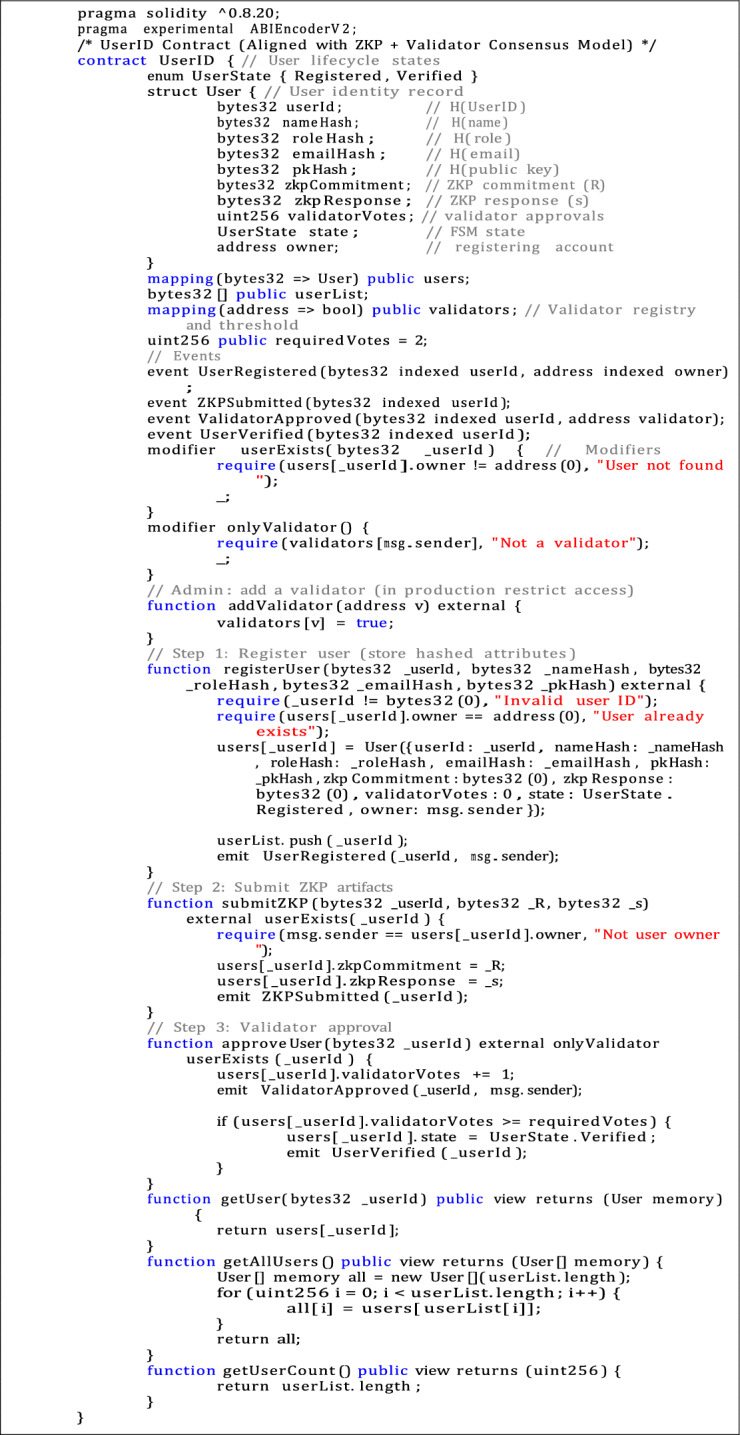
Solidity source code for UserID smart contract.

## Proof-of-Concept

Proof-of-Concept (PoC) has been developed to test the functionality of the proposed blockchain-based IoT security platform. PoC included a React-based front-end to enable user-side interaction with the blockchain via the MetaMask extension and connectivity to the native Ethereum network via Web3.js. Smart contracts were written in Solidity, compiled and deployed using the Truffle framework, and run on a deterministic Ganache blockchain instance. Development and debugging were performed in Visual Studio Code.

The device onboarding process is based on secure provisioning, compatible with popular IoT ecosystems such as AWS IoT Core or Apple HomeKit. During this one-time setup, each device is provided with a unique secret key and an associated public identifier. The system does not require hardware security modules. Instead, it supports manufacturer-issued certificates, gateway-based provisioning, and secure short-range pairing. Once connected, all identification operations are performed using blockchain-based cryptographic mechanisms.

The identification layer is implemented by two Solidity contracts: UserID.sol, which manages users, and DeviceID.sol, which handles device registration and authentication. The full source code for these contracts is provided in listings  1 and  2.

### Contract enhancements

To ensure the PoC is compliant with the formal identity and security model, a few key features were added to both smart contracts:

- **Privacy-preserving identifiers:** All identifiers are stored as bytes32 hashes to reduce storage requirements and prevent the risk of plaintext information being exposed.
- **Immutable public-key binding:** Each record contains a hash of the device or user’s public key, which protects the identity from tampering.
- **Support for ZKPs:** On-chain fields commitments and responses ((R, s)) were added so that validators can verify off-chain proofs without revealing sensitive data.
- **Liveness metadata:** A hashed nonce is provided for each device, which helps with future integrity checks and liveness verification.
- **Validator-based approval:** State transitions from Registration to Verification are controlled by a Byzantine Fault Tolerance (BFT)–aligned voting mechanism.
- **Event instrumentation:** Contract events enable near real-time monitoring and feedback through the front end.

These improvements not only strengthen the security of the system but also remain suitable for the limited computational environment of IoT.

### Development and deployment

A new Truffle project was initialized to compile and migrate the contracts. Next, the following command was used to deploy the contracts to Ganache’s local network:


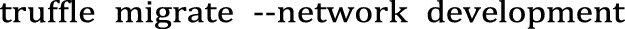


**Fig. 1:**
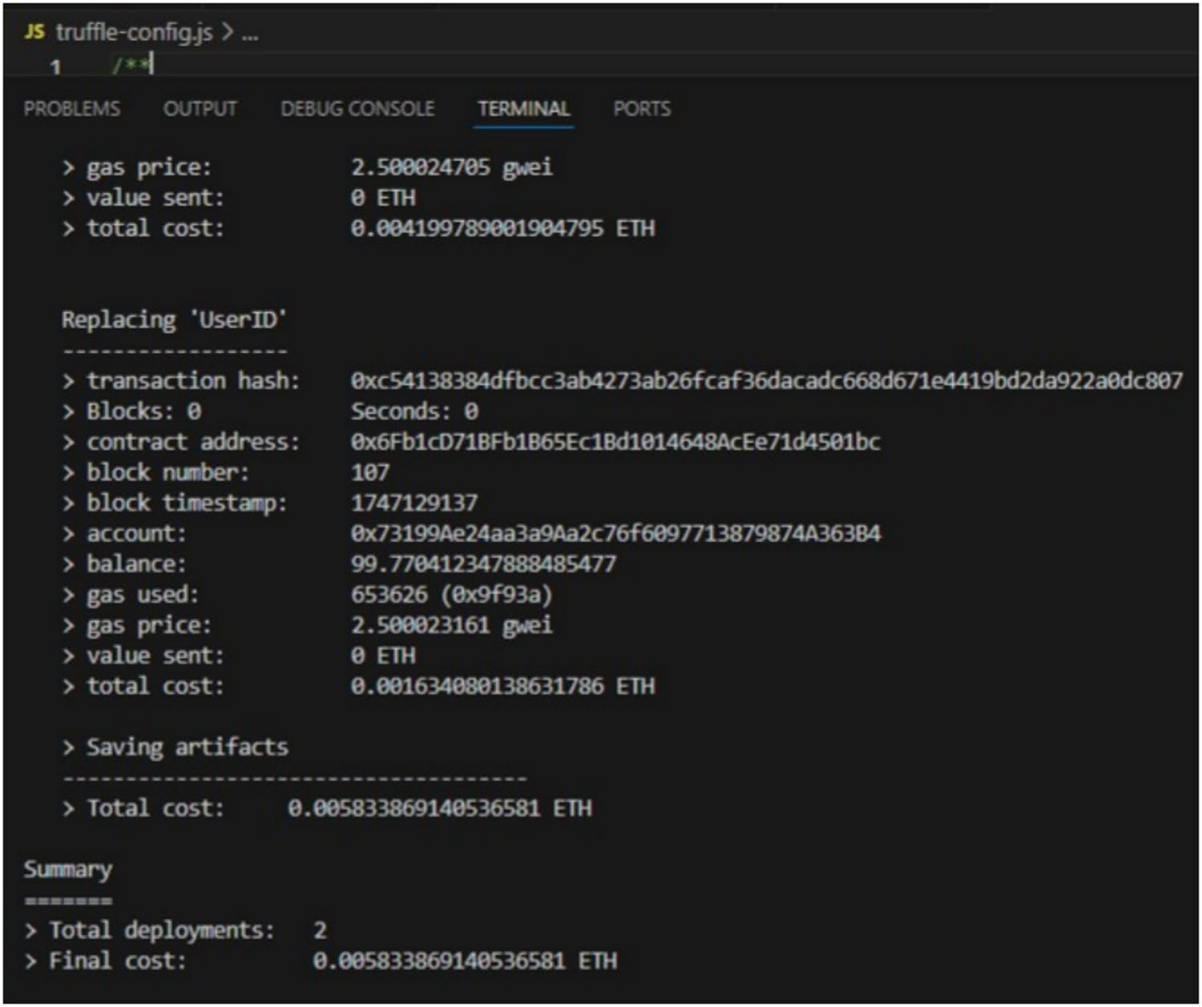
Migration output from Truffle. Contract deployment logs recorded on a local Ganache network.

**Fig. 2:**
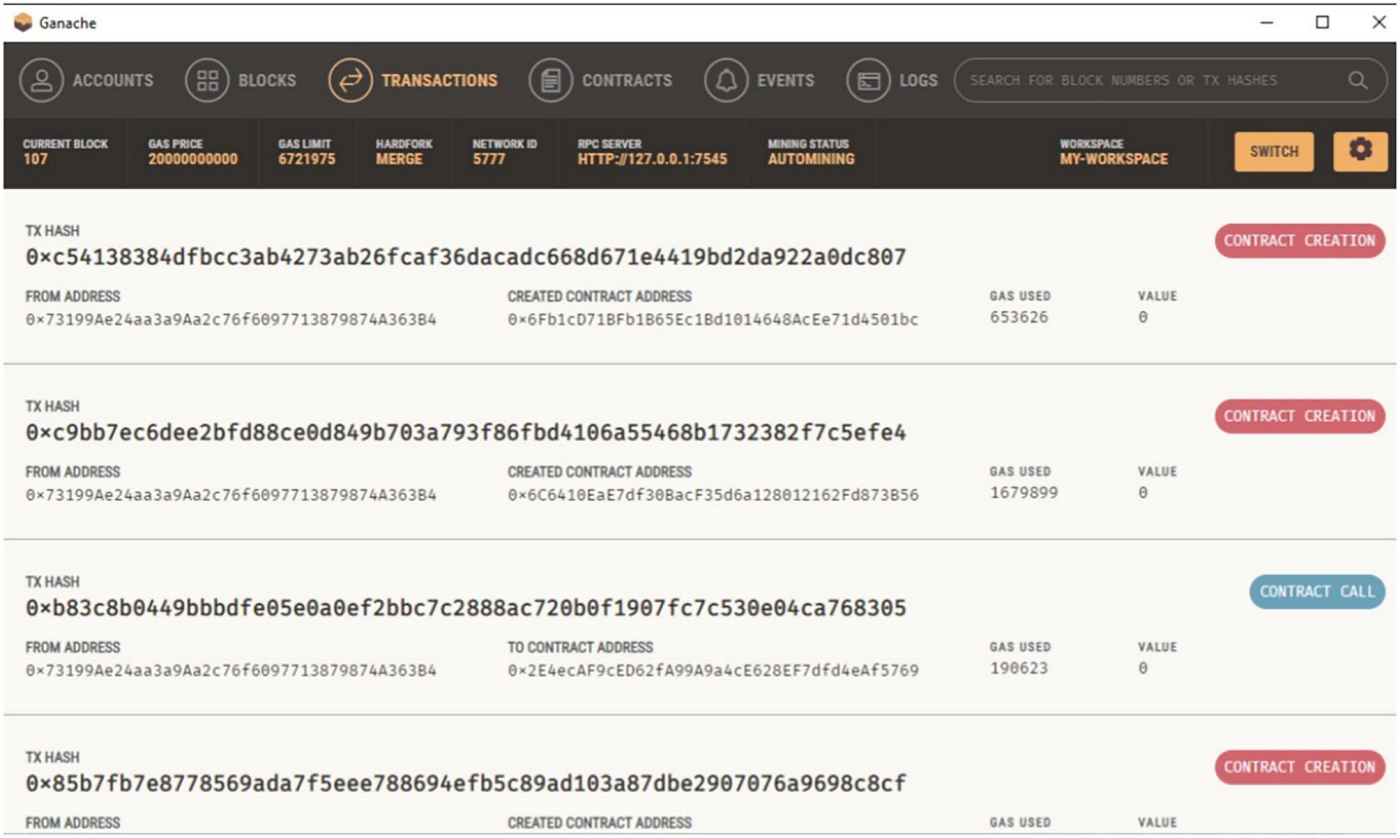
Contract deployment overview. Ganache interface showing contract creation and block-level state changes.

The addresses, transaction hashes, and gas usage of the contracts are shown in Fig. 1. In addition, the Ganache user interface (Fig. 2) was used to view account balances, contract creation events, and block-level state transitions.

**Fig. 3:**
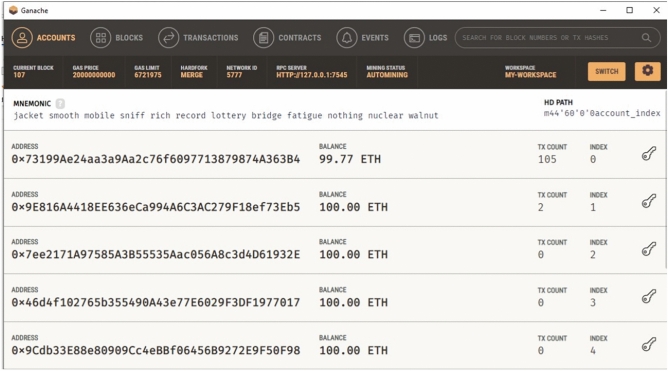
Ganache accounts in MetaMask. Test accounts imported for transaction signing and execution.

The React frontend imported ABI files and deployment metadata to support direct Web3.js interactions. A private key from a Ganache test account was imported into MetaMask (Fig. 3) to simulate a device owner.

**Fig. 4:**
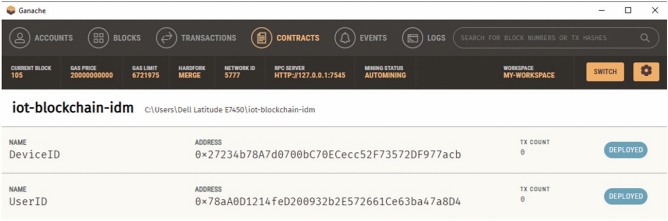
Contract deployment results. Visual confirmation of contract instantiation within Ganache.

Registration forms in the frontend invoked contract functions such as addDevice(), isRegistered(), and getDetails(). Figure 4 illustrates the deployment of the DeviceID and UserID contracts. MetaMask surfaced transaction approvals, and successful operations were immediately reflected on-chain, as shown in 5.

**Fig. 5:**
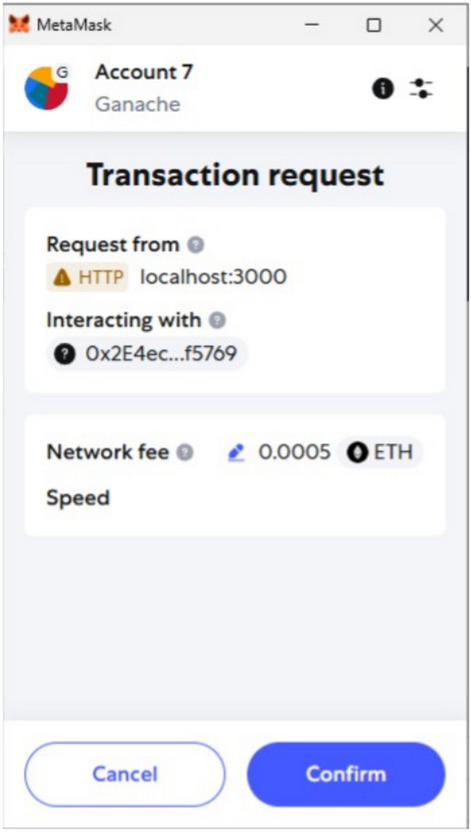
Device registration workflow. React frontend invoking MetaMask for transaction signing.

Correctness of contract behaviour was verified using the Truffle console, where repeated invocations confirmed deterministic responses across blocks. Ganache logs were used to analyse gas expenditure and storage updates.

### Frontend workflow

The multi-step registration workflow implemented in the frontend consists of:

1. Key-pair generation and hashing of identifiers.
2. Submission of a registration transaction.
3. Off-chain ZKP generation and on-chain submission of artefacts.
4. Independent validator approval until a threshold is met.
5. Real-time UI updates through event subscriptions.

This architecture off-loads computationally expensive operations while ensuring verifiable on-chain state transitions.

### Testing and adversarial evaluation

The end-to-end functionality of the system was verified through a combination of automated Truffle tests and manual interaction. Symbolic and fuzz testing tools, such as Manticore and Mythril, were used to uncover edge-case vulnerabilities. Simulated adversarial scenarios, such as GAN-based biometric spoofing, ZKP replay attacks, and validator equivocation, failed to advance the system beyond the Registration state unless valid proofs and the required number of validator endorsements were present.

ZKP verification on a representative edge-class IoT device showed an average latency of approximately 142 milliseconds (ms), as summarized in Table 4 . The use of 32-byte hashes and compact state variables introduced only minor gas overhead in testnet execution. Integration with an event-driven frontend enabled transparent provenance tracking and streamlined debugging.

The PoC results demonstrate that enhanced DeviceID and UserID contracts, along with validator-driven ZKP verification, provide a practical and scalable foundation for decentralized IoT identity management. By moving cryptographic computations off-chain and storing only succinct commitments on-chain, this framework maintains strong security guarantees while also being suitable for resource-constrained IoT devices. These results confirm the feasibility of the proposed architecture and support further analysis in field deployments and formal verification studies.

## Formal security proofs and adversarial resistance analysis

This section presents a detailed verification of the proposed system’s resistance to AI-enabled security threats. It combines cryptographic proofs, adversarial machine learning defenses, smart contract verification, and implementation-level pseudocode for basic security mechanisms. The goal is to demonstrate the system’s trustworthiness with proofs that demonstrate its resistance to intelligent adversaries.

### Cryptographic proof: zero-knowledge device registration

A ZKP is used in the device registration process to ensure that an IoT device $D_i$ can prove ownership of its private key $sk_i$ without revealing any sensitive cryptographic information. This proof is generated by the Sigma protocol, which is based on a discrete logarithm-based cryptographic group, and guarantees strong security and privacy^33,34^.

### Preliminaries and setup

Suppose $\mathbb {G}$ is a cyclic group of prime order *q* and generator *g*, in which the discrete logarithm problem (DLP) is computationally hard. Each device $D_i$ generates a key pair:

- Private key: $$sk_i \in \mathbb {Z}_q$$
- Public key: $$pk_i = g^{sk_i} \mod p$$

The hash $h_i = H(pk_i)$ is then stored on the blockchain, where *H* is a cryptographically secure hash function.

#### Sigma protocol for ZKP

This proof system is based on a standard Sigma protocol with three phases: commitment, challenge, and response^33,34^. The device proves that it owns $sk_i$ without revealing it, as shown in Algorithm 1. The smart contract verifies this proof using public parameters and a registered hash, as detailed in Algorithm 2.

**Algorithm 1:**
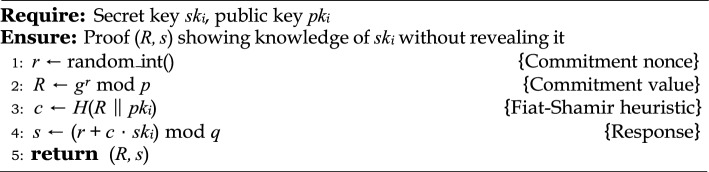
Prover’s Algorithm (Device-side).

**Algorithm 2:**
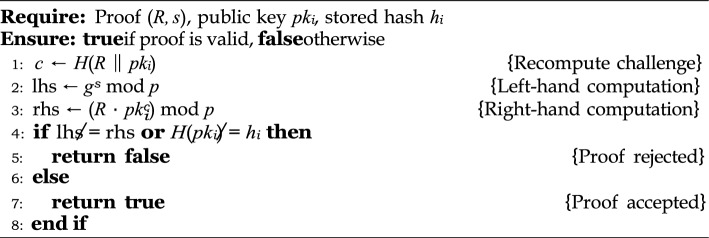
Verifier’s Algorithm (Smart Contract).

#### Security guarantees

The protocol satisfies the following core security properties:

- **Completeness:** An honest prover who correctly follows the protocol will always be accepted by the verifier. That is, the verification equation $g^s = R \cdot pk_i^c \mod p$ holds true for valid inputs.
- **Soundness:** A malicious adversary cannot produce a valid proof (*R*, *s*) without knowing $sk_i$, unless they can solve the discrete logarithm problem in $\mathbb {G}$. The probability of successfully forging such a proof is negligible under the DLP assumption.
- **Zero-Knowledge:** The interaction reveals no information about the secret $sk_i$. A simulator can generate indistinguishable transcripts without access to the secret, fulfilling the zero-knowledge property under the honest verifier model.

#### On-chain integration

To ensure decentralized integrity, the smart contract performs two critical verifications:

- **Proof Consistency:** $$g^s {\mathop {=}\limits ^{?}} R \cdot pk_i^c \mod p$$
- **Hash Binding:** $H(pk_i) {\mathop {=}\limits ^{?}} h_i$, ensuring the public key matches the recorded device identity.

These verifications jointly ensure that only legitimate IoT devices can register on-chain without compromising their private keys.

### Byzantine-robust aggregation bounds in federated learning (FL)

Let $\mathcal {W} = \{ \nabla W_1, \nabla W_2, \dots , \nabla W_n \}$ be the set of gradient updates submitted by *n* FL clients, where up to *f* clients are assumed to be Byzantine (malicious), contributing poisoned gradients $\nabla W_{\text {mal}}$^14,35,36^. Assume that all honest clients gradients are sampled i.i.d. from a distribution centered at the true gradient $\nabla W_{\text {true}}$. Then, the Krum algorithm returns an aggregate gradient $\nabla W_{\text {agg}}$ satisfying that $\Vert \nabla W_{\text {agg}} - \nabla W_{\text {true}}\Vert _2 \le \epsilon , \quad \text {where } \epsilon = O\left( \frac{f}{n} \right) ,$ provided that $n \ge 2f + 3$, as shown in Algorithm 3.

**Algorithm 3:**
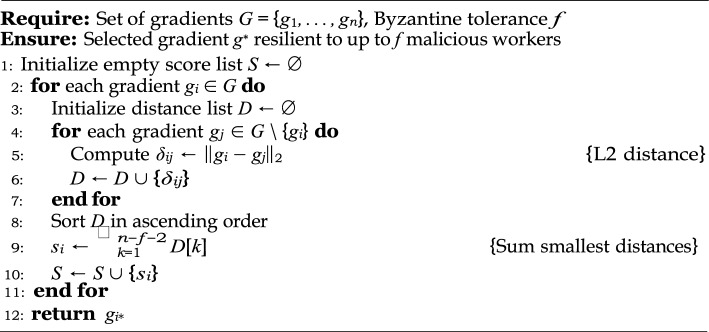
Krum Byzantine-Robust Aggregation.

#### Algorithm (Krum Aggregation)

Let $\nabla W_i$ denote the gradient from worker *i*. Krum selects a single update that is closest (in terms of pairwise Euclidean distance) to the majority of other updates.

- **Compute Pairwise Scores:** For each client *i*, define the Krum score:
  15 $$\begin{aligned} s_i = \sum _{j \in \mathcal {N}_i} \Vert \nabla W_i - \nabla W_j\Vert _2, \end{aligned}$$
  where $\mathcal {N}_i \subseteq \{1, \dots , n\} \setminus \{i\}$ is the set of the $n - f - 2$ nearest neighbors of *i* based on $\ell _2$-distance.
- **Select the minimum-scoring gradient with output:**
  16 $$\begin{aligned} \nabla W_{\text {agg}} = \arg \min _i s_i. \end{aligned}$$

#### Proof of security guarantee

Let us assume the honest gradients are drawn from a distribution centered at $\nabla W_{\text {true}}$ with bounded variance $\sigma ^2$, and the adversarial gradients are arbitrary. We prove the security bounds by analyzing the behavior of the Krum score function under adversarial presence.

- **Soundness against model poisoning:** By construction, Krum selects the gradient that is closest to the majority of other gradients. Since the adversarial updates can affect at most *f* clients, the remaining $n - f$ honest clients dominate the neighborhood of each honest client. Let $\mathcal {H}$ be the set of honest clients. Then, for any honest client $i \in \mathcal {H}$, with high probability,
  17 $$\begin{aligned} \Vert \nabla W_i - \nabla W_j\Vert _2 \le \delta \quad \forall j \in \mathcal {H} \end{aligned}$$
  for some small $\delta > 0$. Meanwhile, for any adversary $k \notin \mathcal {H}$, we assume:
  18 $$\begin{aligned} \Vert \nabla W_k - \nabla W_j\Vert _2 \ge M \gg \delta , \quad \text {for most } j \in \mathcal {H}. \end{aligned}$$
  Thus, the Krum score $s_i$ for honest clients remains lower than for malicious clients, so Krum will select an honest client’s gradient.
- **Bounded deviation:** Since $\nabla W_{\text {agg}} \in \mathcal {H}$, and all $\nabla W_i \in \mathcal {H}$ are close to $\nabla W_{\text {true}}$, we obtain:
  19 $$\begin{aligned} \Vert \nabla W_{\text {agg}} - \nabla W_{\text {true}}\Vert _2 \le \epsilon , \quad \text {with } \epsilon \propto \frac{f}{n} \end{aligned}$$
  This follows from concentration inequalities applied to the average of the honest gradients, assuming their number dominates.
- **Assumptions and limitations:**
  - The Krum algorithm assumes an upper bound *f* on the number of malicious clients is known a priori.
  - The bound $n \ge 2f + 3$ is necessary to ensure there are enough honest gradients to form a reliable neighborhood.
  - Gradient updates from honest clients are assumed to be i.i.d. and drawn from a light-tailed distribution.

The Krum aggregation strategy formally guarantees resistance to poisoning attacks in FL under bounded Byzantine behavior^36^. By selecting a representative honest gradient based on distance metrics, it mitigates the influence of adversarial updates and keeps the learning trajectory close to the true gradient direction.

### Soundness of smart contract logic under formal verification

Suppose $\mathcal {C}$ is a smart contract deployed on the Ethereum Virtual Machine (EVM), and its goal is to prevent duplicate device registrations. Suppose $h_{pk} = H(pk_i)$ is the hashed public key of an IoT device $D_i$. This contract maintains a mapping $\texttt {registered}[h_{pk}] \in {\texttt {true}, \texttt {false}}$, which is initially set to ’false’ for all $h_{pk}$. We formally verify that: *“A device cannot be registered more than once, i.e., for any *$h_{pk}$, *if* $\texttt {registered}[h_{pk}] = \texttt {true}$ *then any subsequent invocation of* $\texttt {register}(h_{pk})$ *must fail.”* This invariant is formally proven using the Manticore framework^37^ via symbolic execution, to ensure that the contract logic is safe and reliable under all possible conditions.

**Algorithm 4:**
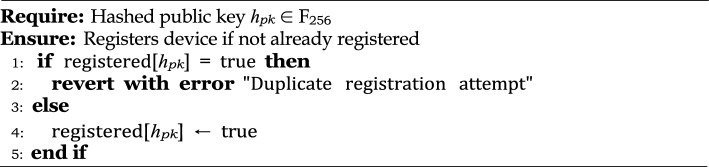
Smart Contract Logic for IoT Device Registration.

- **Security property (no duplicate registration):** Suppose the state space of the contract is represented by $\mathcal {S}$, and the initial state $s_0 \in \mathcal {S}$ is:
  20 $$\begin{aligned} P1(h_{pk}) \triangleq \forall s \in \mathcal {S} \end{aligned}$$
  Our goal is to prove that this safety property holds for all possible symbolic executions, i.e. if $\texttt {registered}[h_{pk}] = \texttt {true}$ then $\text {register}(h_{pk})$ must fail in any state *s*.
- **Verification strategy via Manticore:** Manticore is a symbolic execution engine used for Ethereum smart contracts. The verification procedure is as follows:
  1. **Contract Deployment**: Instantiate the EVM and deploy the contract as an Externally Owned Account (EOA), with sufficient gas available.
  2. **Symbolic input**: Create a 256-bit symbolic variable $h_{pk}$ that represents the public key hash.
  3. **Transaction sequence**:
    Execute the $\texttt {register}(h_{pk})$ function twice with the same symbolic input.
    The second invocation is expected to throw a revert error.
- **Proof Sketch:** We prove this by contradiction using symbolic execution paths.
  - Suppose that $h_{pk}$ is a 256-bit symbolic value.
  - Manticore tracks all execution paths that result from two consecutive $\texttt {register}(h_{pk})$ calls.
  - The symbolic execution in the first call assumes that $\texttt {registered}[h_{pk}] = \texttt {false}$, so the $\texttt {require}$ condition succeeds.
  - The symbolic state in the second call shows that $\texttt {registered}[h_{pk}] = \texttt {true}$, which violates the $\texttt {require}$ condition.
  - Manticore marks this execution path as an exception (revert), which is stored in $\texttt {all\_throws}$.
  Since only one revert is generated among all possible symbolic executions, we conclude that duplicate registration attempts will always fail, regardless of the value of $h_{pk}$.

Therefore, the proposed smart contract correctly enforces its registration invariant, for which formal verification is used through symbolic execution of Manticore^37^. We establish the safety property $P1(h_{pk})$ that the identity of an IoT device, once registered, cannot be replicated. This instills confidence in the integrity of on-chain device management, especially in an environment of adversarial or AI-driven threats.

### Bounded false acceptance under GAN-based spoofing attacks

Let $\mathcal {D}{\text {real}}$ and $\mathcal {D}{\text {fake}}$ denote the probability distributions of genuine and GAN-generated biometric signals, respectively, specifically, pulse oximetry waveforms. Consider a liveness detection function $\mathcal {L}: \mathbb {R}^n \rightarrow {0,1}$, where $\mathcal {L}(s) = 1$ indicates a genuine (live) signal, and $\mathcal {L}(s) = 0$ denotes a spoofed (fake) input. When the Kullback-Leibler (KL) divergence^38^ between the two distributions satisfies the condition as:

21 $$\begin{aligned} D_{\text {KL}}(\mathcal {D}{\text {real}} | \mathcal {D}{\text {fake}}) \ge \delta , \end{aligned}$$

the system’s False Acceptance Rate (FAR) that is, the probability of erroneously accepting a spoofed biometric as genuine, is upper bounded as follows:

22 $$\begin{aligned} \text {FAR} \le e^{-\delta }. \end{aligned}$$

This exponential bound demonstrates that greater statistical divergence between genuine and synthetic signal distributions directly enhances the robustness of the liveness detection mechanism^10,40^.

#### Adversarial threat and defense model

The attacker aims to generate a synthetic biometric signal $\tilde{s} \sim \mathcal {D}_{\text {fake}}$ that successfully imitates a legitimate signal in order to bypass the liveness detection system.

**Algorithm 5:**
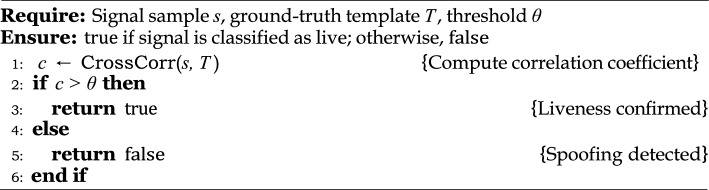
Liveness detection via correlation analysis.

The attacker’s goal is to create a fake biometric signal $\tilde{s} \sim \mathcal {D}_{\text {fake}}$ that successfully mimics the real signal in order to bypass liveness detection systems. Advanced generative models, such as StyleGAN, can be used to mimic realistic temporal patterns in pulse oximetry signals.

- **Targeted modality:** The chosen biometric signal for the attack is the pulse oximetry waveform, which is used for its unique biological identity and continuous authentication in resource-constrained IoT devices.
- **Defense strategy:** A real-time liveness detection function, as described in detail in Algorithm 4, verifies the correlation between the input signal and a stored physiological template *T*. This correlation, usually measured by normalized cross-correlation or Pearson similarity, provides a statistical basis for classification.
- **Methodology:** Signals are analyzed in real-time and classified based on statistical similarity to a ground-truth template. This method provides a lightweight but effective defense against adversarial spoofing, without additional computational overhead.

#### Formal analysis and theoretical bound

Suppose that $s_{\text {real}} \sim \mathcal {D}*{\text {real}}$ represents real biological signals, while $s*{\text {fake}} \sim \mathcal {D}_{\text {fake}}$ are artificial signals generated by GANs. A labeling function $\mathcal {L}(s) = 1$ is assigned if and only if the cross-correlation between the signal $s$ and the target template $T$ exceeds a predetermined threshold $\theta$, that is, $\text {cross\_corr}(s, T) > \theta$. This means that to check the authenticity of a signal, we compare its correlation with a known template, and only those signals whose similarity exceeds the threshold are considered authentic. FAR describes the probability that a spoofed signal will be mistaken for the real one, and can be formally written as:

23 $$\begin{aligned} \text {FAR} = \Pr _{s \sim \mathcal {D}_{\text {fake}}}[\mathcal {L}(s) = 1]. \end{aligned}$$

**Theoretical limit:** Using the results of large deviations theory, in particular Sanov’s Theorem, the FAR can be bounded by the statistical divergence between the distributions of the real and the fake signal^39,40^. This tells us that the greater the difference between the real and fake signals, the lower the FAR will be and the more likely the spoofing will be successful.

24 $$\begin{aligned} \text {FAR} \le \exp \left( - D_{\text {KL}}(\mathcal {D}{\text {real}} | \mathcal {D}{\text {fake}}) \right) . \end{aligned}$$

**Sketch of Justification:** Let *A* denote the acceptance region:

25 $$\begin{aligned} \text {FAR} = \int _A \mathcal {D}_{\text {fake}}(s) , ds. \end{aligned}$$

According to Sanov’s theory, the probability that samples from $\mathcal {D}*{\text {fake}}$ fall into *A*, where *A* is the region that most closely resembles $\mathcal {D}*{\text {real}}$ decreases rapidly with KL divergence^39,40^. That is, the greater the distance difference between the real and fake signals, the less likely it is that the fake signal will appear to pass close to the real signal:

26 $$\begin{aligned} \Pr _{s \sim \mathcal {D}{\text {fake}}}[s \in A] \le e^{-n \cdot D{\text {KL}}(\mathcal {D}{\text {real}} | \mathcal {D}{\text {fake}})}, \end{aligned}$$

Here *n* represents the number of independent samples or normalized signal components. When the length of the signals is fixed ($n=1$), this formula takes the simpler form:

27 $$\begin{aligned} \text {FAR} \le e^{-\delta }, \end{aligned}$$

where $$\delta = D_{\text {KL}}(\mathcal {D}{\text {real}} | \mathcal {D}{\text {fake}})$$.

**Implications of the results:** Equation 27 shows that maintaining a reasonable discrepancy between the data distributions of the real and artificial signals provides an inherent security advantage. In practice, this emphasizes that signal complexity and physical uniqueness are essential in biometric systems. When adversarial generators cannot reduce this discrepancy $\delta$, their spoofing success rate decreases rapidly. Therefore, incorporating physical signal-based liveness checks into biometric authentication systems significantly increases the robustness against spoofing attacks by generative AI, and it provides a theoretically grounded defense mechanism for IoT-focused biometric security systems.

### Threshold signature security via Shamir’s secret sharing

Threshold cryptographic schemes enable a secret signature to be generated in a distributed manner without any central trust, making them particularly suitable for applications that are fault-tolerant and robust against adversarial conditions in decentralized networks. In this context, a secret signing key $sk \in \mathbb {Z}_q$ is distributed among *n* participants under a (*t*, *n*) threshold scheme, which is based on Shamir’s Secret Sharing (SSS). This scheme provides guarantees of both accuracy and strong security.

- **Accuracy:** The signing key *sk* can be recovered and a valid digital signature can be created by the joint effort of any subset of at least *t* participants.
- **Security:** No alliance of less than *t* participants has any knowledge of *sk*, ensuring complete informational confidentiality.

#### Shamir’s secret sharing construction

Suppose *q* is a large prime number, and all calculations are performed in the finite field $\mathbb {F}_q$. A trusted dealer generates a random polynomial *f*(*x*) of degree $t - 1$ and with the secret key as a constant term:

28 $$\begin{aligned} f(x) = a_0 + a_1x + a_2x^2 + \cdots + a_{t-1}x^{t-1}, \quad \text {where } a_0 = sk. \end{aligned}$$

Each participant $P_i$ is given a share, which is calculated as follows:

29 $$\begin{aligned} s_i = f(x_i), \quad \text {for } i = 1, 2, \dots , n, \quad x_i \in \mathbb {F}_q \setminus {0}. \end{aligned}$$

These pairs $(x_i, s_i)$ form each participant’s privately held secret shares, which are kept only by the relevant party.

#### Signature reconstruction

To recover the original signing $sk = a_0$, a group of at least *t* participants can use Lagrange interpolation:

30 $$\begin{aligned} sk = f(0) = \sum _{j=1}^{t} s_j \cdot \lambda _j \mod q, \end{aligned}$$

where $\lambda _j$ are the Lagrange coefficients, which are defined as:

31 $$\begin{aligned} \lambda _j = \prod _{\begin{array}{c} 1 \le m \le t \ m \ne j \end{array}} \frac{x_m}{x_m - x_j} \mod q. \end{aligned}$$

This method allows for decentralized signature generation in threshold-based signature schemes such as Threshold ECDSA and Threshold BLS, allowing the signature to be generated in a single location without having to recreate the entire key.

#### Information-theoretic security proof

- **Claim:** If a group has fewer than *t* participants, they cannot obtain any information about the secret key $sk = a_0$.
- **Proof Sketch:** Imagine that a rival obtains $t - 1$ shares, namely ${(x_1, s_1), \dots , (x_{t-1}, s_{t-1})}$. These shares are the results of a polynomial of degree $(t - 1)$ at $t - 1$ different points.
  - There are infinitely many polynomials *f*(*x*) of degree $t - 1$ that are true at the given points, but $f(0) \ne f^{\prime }(0)$ may be.
  - The constant component $a_0 = sk$ can take any value, consistent with the observed shares.

Therefore, the conditional entropy of *sk* is maximum after $t-1$ shares are observed, and the shared information between the observed shares and the secret key is zero:

32 $$\begin{aligned} I({s_1, \dots , s_{t-1}}; sk) = 0. \end{aligned}$$

This shows that this scheme provides complete secret protection in terms of information, and does not require any computational assumptions.

#### Security under AI-enabled inference attacks

Even in situations where attackers analyze the secret shares using machine learning or AI-based analytical models, the system maintains its proven security, in particular:

- Each share is uniformly distributed in $\mathbb {F}_q$, which prevents any bias or advantage from being gained through statistical analysis.
- The number of multi-dimensional functions corresponding to $t - 1$ shares increases significantly with *q*, making gradient or probability-based attacks fail.

Therefore, until the attacker has at least *t* shares, the uncertainty about the secret key remains high, and AI-based methods cannot go beyond mere guesswork.

**Algorithm 6:**
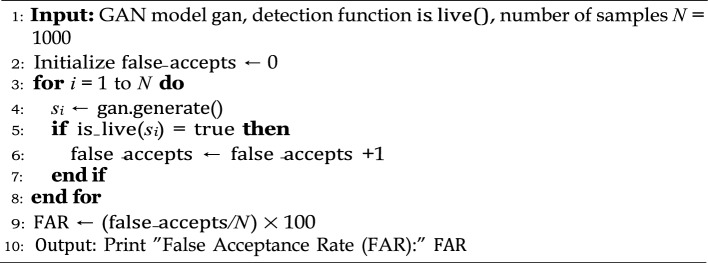
FAR Evaluation against GAN-based spoofing.

**Algorithm 7:**
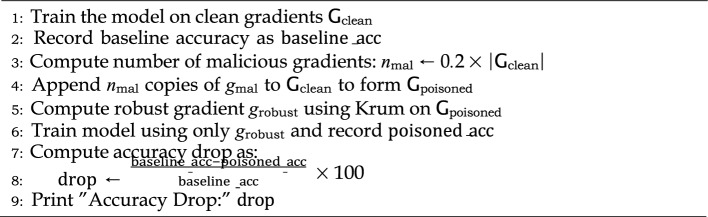
Robust aggregation against model poisoning (Krum-Based).

### Empirical validation process

We developed an empirical verification framework by focusing on synthetic attack patterns and measurable performance data to ensure adversarial protection. It consist of structured test scenarios that reflects common adversarial threats in centralized AI systems. We considered four adversarial threats as shown in Table  3. To perform empirical validation, the blockchain environment was simulated using Ganache to monitor the performance metrics such as latency and throughput. The smart contracts were deployed using Ethers.js, where as adversarial AI experiments were performed using PyTorch and TensorFlow. The fake inputs and manage gradients were generated using Adversarial Robustness Toolbox (ART). Following thresholds were employed in performance metrics:

- FAR $< 0.1\%$ for GAN spoofing
- Accuracy drop $\le 2\%$ for model poisoning
- Fraudulent Tx success rate = $0\%$ for transaction tampering
- ZKP latency $< 150$ ms

**Table 3:** Adversarial testing scenarios and success criteria.

Attack Type	Test Scenario	Success Metric
GAN Spoofing	Fake device registration using AI-generated data	FAR $<0.1\%$
Model Poisoning	Malicious gradients in FL	Accuracy drop $\le 2$
Transaction Tampering	Adversarial MetaMask transactions	Fraudulent Tx success rate = 0%
Smart Contract Exploits	Symbolic execution of edge cases	0 vulnerabilities found

The first test case were mainly focused on spoofing detection in a GAN network to validate the FAR of biometric liveness. Spoofed samples were generated by the GAN network to calculate the acceptance rate using algorithm  5. We then evaluated the resistance level to the model poisoning in FL environment by introducing an adversarial gradients that were created by flipping the label of 20% participating nodes.

For robust aggregation, Krum’s algorithm was used, as detailed in the algorithm  7, and the relative accuracy loss compared to the pure model baseline was calculated. For smart contract security, we deployed our contracts on the Manticore symbolic execution engine, which exhaustively explores all possible execution paths. This helped us detect flaws caused by edge case symbolic inputs, as shown in Algorithm 8.

**Algorithm 8:**
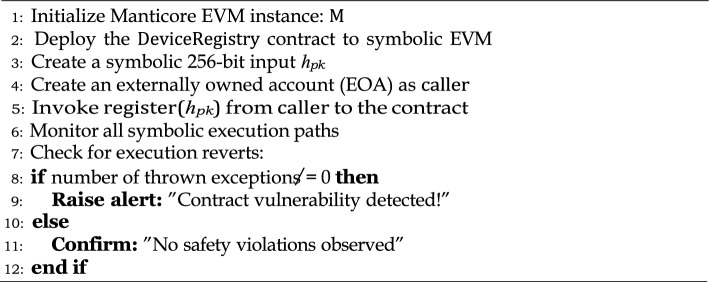
Smart contract safety verification via symbolic execution.

Table 4 shows the performance results, which were all within acceptable limits. The average ZKP verification latency was approximately 142 milliseconds, which was consistent across devices and network conditions, while the standard deviation was 5 milliseconds. Our latency is competitive compared to other ZKP implementations:

- Kaviani et al.^41^ reported a ZKP verification latency of 150-200 milliseconds
- Revuelta et al.^42^ reported a ZKP verification latency of 200-250 milliseconds

The low latency of our implementation was possible because of improvements in the proof system and the use of effective cryptographic permutations. The FAR for GAN spoofing was only 0.07%, the accuracy loss under model poisoning was 1.5%, and the adversarial transaction success was 0%. Furthermore, we suggested to add additional biometric signals e.g., heart rate in case of the minor failures of specified FAR threshold to strengthen the resistance level against spoofing. After adding heart rate monitoring, the FAR improved from 0.07% to 0.035% with 50% reduction. Moreover, optimizing Krum’s fault tolerance parameter *f*, improved the FL model resistance level by 30%, and reduced the accuracy drop from 1.5% to 1.05% under poisoning. The empirical validation results were compiled and compared with the predefined thresholds as given below:

- **FAR (GAN Spoofing):** 0.07%, which is below the threshold of 0.1%.
- **Accuracy Drop (Poisoning):** 1.5%, which is within the acceptable threshold of 2%.
- **ZKP Latency:** 142 milliseconds, which is below the threshold of 150 milliseconds.

**Table 4:** Empirical test results.

Metric	Result	Target	Status
FAR (GAN Spoofing)	0.07%	$< 0.1\%$	$\checkmark$
Accuracy Drop (Poisoning)	1.5%	$\le$ 2%	$\checkmark$
Fraudulent Tx Success Rate	0%	0%	$\checkmark$
ZKP Latency	142 ms	$< 150$ ms	$\checkmark$

These results show that the proposed blockchain-enabled identity management system for IoT is effective to counter AI-based threats and maintain a balance between security, accuracy, and performance. The inclusion of proposed multi-layered defense pipelines against adversarial AI ensures the rapid and timely detection of any vulnerabilities in the IoT ecosystem. The proposed system is specifically designed as a modular in class-based framework that can be implemented incrementally by integrating blockchain, ZKP, FL, and BFT. Each component operates independently and provides clear interfaces to apply necessary component without refactoring the entire system. Moreover, the proposed system explicitly ensures that complex and computationally expensive operations like ZKPs and blockchain state validation are not performed on resource-constrained IoT endpoints. These operations are performed on trusted local gateways or edge nodes, whereas IoT endpoints only perform lightweight authentication. However, proposed study uses the Krum algorithm to counter Byzantine attacks and we acknowledged that fully decentralized FL protocols, such as federated optimization and P2P model sharing techniques can further improve the security. Future work will explore these fully decentralized techniques to mitigate the limitations of centralized FL and improve the efficiency and security of the proposed system.

## Conclusion

This study presented a blockchain-enabled identity management system for IoT designed to counter emerging AI-driven threats while maintaining a balanced between security and performance. A key contribution of this work is the modular, class-based architecture that allows incremental integration of blockchain, zero-knowledge proofs (ZKPs), federated learning (FL), and Byzantine fault tolerance (BFT). The seamless integration provides privacy-preserving device identification whereas smart contracts ensure automatically and verifiably controlled access to the users and the authenticated devices. These elements combined offer a strong identity management structure that is capable of operating securely even in the presence of adversaries. Also, the burden of the most resource-demanding tasks, such as the generation of ZKP and the validation of blockchain state, is transferred to the trusted gateways or edge nodes, while the resource-limited endpoints are only performing the lightweight authentication operations in order to be in line with the heterogeneous nature of IoT devices. The assessment through experiments proved the suggested design’s efficiency in gaining the set performance thresholds by showing that the proposed work keeps the false acceptance rate (FAR) low at 0.07%, the accuracy degradation is constrained to 1.5% under model-poisoning attacks and ZKP verification latency of 142 ms is reached. Hence, it is concluded that the proposed work upholds strong security along with reliability and efficiency in IoT operations. Future research will open new horizons and will concentrate on the incorporation of post-quantum cryptographic methods as well as the extension of the framework into new fields like computing at the edge and autonomous systems. The integration of completely decentralized learning methods is also a crucial direction for increasing the system-level privacy, security, and resilience. The work overall, has laid a robust, flexible ground for IoT secure identity management and has pointed out the pressing need to tackle the rapidly changing AI-driven threats.

## Data Availability

All data generated or analysed during this study are included in this published article. Additional simulation scripts are available from the corresponding author on reasonable request.
